# Antimicrobial characteristics of Berberine against prosthetic joint infection-related *Staphylococcus aureus* of different multi-locus sequence types

**DOI:** 10.1186/s12906-019-2558-9

**Published:** 2019-08-16

**Authors:** Jiaqi Tan, Jin Wang, Chuang Yang, Chongzun Zhu, Geyong Guo, Jin Tang, Hao Shen

**Affiliations:** 1 Department of Orthopaedics, Shanghai Jiao Tong University Affiliated Sixth People’s HospitalShanghai Jiao Tong University, Shanghai, 200233 China; 2 Department of Clinical Laboratory, Shanghai Jiao Tong University Affiliated Sixth People’s Hospital, Shanghai Jiao Tong University, Shanghai, 200233 China

**Keywords:** Prosthetic joint infection, Berberine, *Staphylococcal aureus*, Multi-locus sequence types, Biofilm

## Abstract

**Background:**

*Staphylococcal aureus (S. aureus)* has become the leading causative pathogen of Prosthetic Joint Infection (PJI), which is the most devastating complication after arthroplasty surgeries. Due to the biofilm formation ability and emergence of multiple-drugs resistance strains of *S. aureus*, it has become an urgency to find new anti-staphylococcal agents to establish effective prophylaxis and treatment strategy for PJI. Extracted from a traditional Chinese herb, berberine is proved active in inhibiting *S. aureus*, while whether it exerts the same effect on PJI-related *S. aureus* remains unknown. This study aims to investigate the antimicrobial activity of berbrine against clinical derived PJI-related *S. aureus* and whether its inhibiting efficacy is associated with subtypes of *S. aureus*.

**Methods:**

Eighteen PJI-associated *S. aureus* were collected and their Multi-locus Sequence Types (MLST) and susceptibility to berberine both in planktonic and biofilm form were investigated. Additionally, one *S. aureus* strain (ST1792) was selected from the group and its transcriptomic profiling in berberine incubation was performed. The statistical analyses were conducted using Student’s t-test with SPSS 24.0(SPSS, IBM, USA). The data were expressed as the means ± standard deviation. Values of *p* < 0.05 were considered statistically significant.

**Results:**

It was found out that the Minimum Inhibitory Concentration values of PJI-related *S. aureus* varied in a broad range (from 64 to 512 μg/ml) among different MLST subtypes and the bacteria were able to regain growth after 24 h in berberine of MIC value or higher concentrations. In addition, sub-inhibitory concentrations of berberine surprisingly enhanced biofilm formation in some *S. aureus* strains.

**Conclusion:**

Traditional medicine is utilised by a large number of individuals, which provides abundant resources for modern medical science. In our study, berberine was found bactericidal against PJI related *S. aureus*, however, its antibacterial property was impacted by the MLST subtypes of the bacteria, both in planktonic and biofilm growth forms.

## Background

Prosthetic joint infection (PJI) is a disastrous complication after arthroplasty surgeries which causes great burden to both patients and surgeons, as well as social economy [1–4]. *Staphylococcus aureus (S. aureus)* is one of the leading causative pathogens of PJI [5–8]. Due to its array of virulence and the ability to form biofilm [7, 9–13], *S. aureus*-induced PJI is particularly difficult to combat. Furthermore, in recent years, the increasing occurrence of high morbidity and mortality of PJI has been proved associated with multidrug-resistant *S. aureus* [14, 15]. Therefore, finding new antimicrobial agents to help establish effective treatment and prophylaxis against *S. aureus* associated PJI has become a major concern in the surgeons’ community.

Berberine is an isoquinoline alkaloid extracted from a traditional Chinese herb named Huang-Lian (*Coptis chinensis*) and has been used for decades as an OTC (Over the Counter) drug to treat diarrhea and bowl disorder in China. Studies have found a lot more biological activities of berberine, such as antitumor [16, 17], antidiabetic [18–20], antiviral [21, 22], antifungal [23] and antibacterial in particular [24–28]. Robert et al. tested the antimicrobial effect of berberine against Coagulase-Negative staphylococcus standard strains [29]. Chu et al. demonstrated that berberine is active in killing Methicillin-Resistant *S. aureus* (MRSA) [30]. Guo et al. proved berberine was effective in inhibiting growth of *S. aureus* both in planktonic and biofilm cultures [31]. However, the strains tested in these studies were either not *S. aureus* strains or non-clinical strains. And the clinical *S. aureus* strains tested by Guo et al. were not associated with PJI. Further, the MLST types of the clinical *S. aureus* were not determined while different subtypes of *S. aureus* could have vastly different sensitivity to the same antimicrobial agents. These disadvantages may hinder the way of applying berberine into clinical PJI control. Therefore, in this study, we collected eighteen PJI-associated clinical *S. aureus* strains, determined their MLST types and tested the inhibitory effect of berberine against *S. aureus* in planktonic and biofilm status. Besides, to investigate the underlying mechanisms of berberine inhibiting *S. aureus*, transcriptomic profile of *S. aureus* treated with berberine was investigated by transcriptome sequencing of a clinical PJI-related *S. aureus* strain. And bioinformatics analysis including DEGs (Differentially Expressed Genes) selection, GO (Gene Ontology Consortium) functional enrichment and pathway significance enrichment were conducted.

## Methods

### Bacteria strains, media and reagents

In all, 18 isolates of *staphylococcal aureus* were collected from the cultures of PJI patients in joint surgery department, Shanghai Sixth people’s Hospital, Shanghai, China. After collection, the bacteria were given a series number from A to R and stored in Tryptic Soy Broth (TSB) medium with 20% of glycerol at − 80 °C for further use. Mueller–Hinton broth II (MHB II) and Tryptic soy broth (TSB) were purchased from Sangon Biotech (Shanghai, China). The berberine chloride (C20H18ClNO4, molecular weight 371.81) was purchased from Sigma-Aldrich (St Louis, MO, USA) and stock solutions at various concentrations were made in 1% dimethyl sulfoxide (DMSO) (Sangon Biotech) [32].

### MLST determination and Berberine MICs test

Bacterial MLST was determined by the PCR amplification and sequencing of seven housekeeper genes (*arc, aro, glp, gmk, pta, tpi, yqd*) and referring the results to the database online. The MIC values of berberine against eighteen PJI-related *S. aureus* strains were determined using twofold serial dilutions in MHB II according to CLSI/NCCLS M100-S15 (CLSI, 2005) in triplicate. The MICs were defined as the lowest concentrations at which no visible growth was observed after 24 h.

### Biofilm assay

Berberine was added to the TSB broth containing 1% glucose in 96-well plates (Corning Co., NY, USA) to reach a final volume of 100 μl and a series of concentrations (2, 4, 8, 16, 32, 64, 128, 256, and 512 μg/ml was only made when testing strain ST39). The cultures were then inoculated with 100 μl seed culture of *S. aureus* (5 × 10^5 CFU/mL). After incubating for 24 h at 37 °C, the supernatant was completely removed and the wells were washed three times with phosphate buffered saline (PBS) (pH 7.2). Then 200 μl methanol was used to stabilize the biofilm for 30 min and then dried at 60 °C. After that, the biofilm was stained with 200 μl of 0.1% crystal violet for 15 min. Unbound crystal violet was rinsed by PBS for three times. After drying 200 μl of 95% ethanol was added to each well and the plates were shaken for 1 h to release the stain from the biofilm. The absorbance of the biofilm was measured at 600 nm for three times. Wells containing 1% DMSO and bacteria was the bacterial growth control (GC).

### Growth kinetics test

A suspension of 100 μl bacterial cultures (5 × 10^5 CFU/mL) in TSB was added to 100 μL of serially diluted berberine (0.5, 1, 2, 4, 8, 16, 32, 64, 128, 256, and 512 μg /mL) in 96-well plates (Corning Co., NY, USA). Microplates were incubated at 37 °C for 2, 6, 12 and 24 h, and the bacterial growth was evaluated by measuring the optical density of cultures at 600 nm wavelength with a Multiskan EX microplate reader (Thermo Electron Corp., Vantoa, Finland). Wells Molecules containing 1% DMSO with bacterial inoculum served as the bacterial growth control (GC).

### Treatment with berberine

*Staphylococcus aureus* strain ST1792 was grown overnight in 4 mL of TSB at 37 °C. Two 15 ml test tubes containing 10 mL of TSB were inoculated with an overnight culture with an initial OD600 of 0.05. The bacteria were grown at 37 °C at 220 rpm to an OD600 of 0.3~0.4. Then, 500 μl of 1280 μg/mL berberine stock solution was added to the experiment tube, and DMSO solution was added to the control tube. The final concentration of berberine in the experimental tube was 1/2 MIC (64 μg/ml). The final concentration of DMSO in each culture was 1%, and such amounts of DMSO did not change the pH of the medium. The experimental and control cultures were incubated for a further 45 min at 37 °C, and then bacterial cells were collected and RNA isolation was performed. Three independent experiments were performed.

### Total RNA isolation

The collected bacterial cells were placed in the RNA-protect Bacteria Reagent (QIAGEN GmbH, Germany) and incubated for 5 min at room temperature to stabilize the mRNA. After that, the cell suspensions were centrifuged at 8000×g for 5 min and the supernatant was discarded. Total RNA was purified using RNeasy Mini Kit (Qiagen) according to the manufacturer’s protocol. RNA quantity was measured using Agilent 2100 bioanalyzer.

### Enrichment and sequencing of mRNA

A total of 10 μg of each RNA sample was subjected to further purification to enrich the mRNA using a MICROB Express Kit (Ambion) according to the manufacturer’s instructions. The mRNA sample was suspended in 25 μl RNA storage solution and the quality of mRNA was determined using Agilent 2100 Bioanalyzer. Bacterial mRNA was fragmented and the fragments were achieved in the size range of 200–250 bp using the Illumina TruSeq Stranded Kit (Illumina, USA), which was also used to generate the double-stranded cDNA to prepare RNA-seq library. All of the samples were sequenced using the Hiseq2000 (Illumina, USA) sequencer at Beijing Genomics Institute at Shenzhen.

### Transcriptome assembly and annotation

Reads were aligned to *Staphylococcus aureus subsp. aureus* ST1792 (Genome sequenced before, data not shown) using the HISAT (Hierarchical Indexing for Spliced Alignment of Transcripts) [33]. The RNA-seq data analysis included the following steps. (1) If the pair-end reads satisfied *N* > 2% and low quality (quality value < 20) > 50%, the reads were removed. In addition, if the terminal 20 bp consisted of N or was of low quality, the reads were removed. Clean data were produced using the above quality control (QC) standards. (2) The clean data were aligned to *S. aureus* ST1792 using BWA. (3) Reads that could not be mapped or had incorrect alignment were removed. Quality control (QC) of alignment was produced on the standard above. (4) The commonly used fragments per kilo-base of transcript per million mapped fragments (FPKM) incorporate normalization steps to ensure that expression levels for different genes and transcripts are comparable across runs [34]. Based on FPKM normalization, analyses of distribution, coverage and differentially expressed genes were also performed.

### Identification of differentially expressed genes

Differentially expressed genes were identified using DESeq2, which calculated expression in two or more samples and tested the statistical significance of each observed expression changes between them. Genes with an adjusted *P* value < 0.05, FDR ≤ 0.01 and fold change ≥2 were identified as differentially expressed. Finally, heat map and volcano plot were used to visualize and integrate the data produced by DESeq2 analysis.

### Quantitative real-time RT-PCR used to validate RNA-seq data

Nine genes (*icaA, icaR, fnbA, lrgA, lrgB, cidA, srrB, spa, nuc*) that were significantly differential transcribed (*P* < 0.05, FDR < 0.01) under berberine treatment and related to the production of three types of biofilm matrix were selected for qRT-PCR to validate RNA-seq data. Gene information and primer used were shown in Table 1. RNA was reverse transcribed into cDNA using the PrimeScript™ RT reagent Kit with gDNA Eraser (TaKaRa, Japan) according to the manufacturer’s instructions. The qRT-PCR was performed in a 20 μ L volume using SYBR Green qPCR Master Mix (TaKaRa, Japan) as recommended by the manufacturer. The cDNA was subjected to real-time RT-PCR using the primer pairs listed in Table 2. Cycling conditions were 48 °C for 30 min and 95 °C for 15 min, followed by 40 cycles of 95 °C for 15 s and 60 °C for 1 min, and a dissociation step of 95 °C for 15 s, 60 °C for 30 s, and 95 °C for 15 s. Fold change between treatment samples and controls was calculated using 2–ΔΔCt method. All samples were analyzed in triplicate and housekeeping gene *pyk* was used as the internal reference to obtain basis of normalization [35, 36].

**Table 1:** Primers of quantitation Real-time PCR (qRT-PCR) used in this study

Gene Name	Primer	Sequences (5′–3′)
*icaA*	F	TGCTGGCGCAGTCAATACTA
	R	CATGGCAAGCGGTTCATACT
*icaR*	F	CCTTATTTTCAGAGAAGGGGTATG
	R	CGAATACACTTCATCTTTGAATTG
*fnbA*	F	GTCAAGTTATGGCGACAGGA
	R	GCGTCACTGTTGTAGGATCA
*lrgA*	F	GTCGTGAAACAACAAAAAGACGCA
	R	TTAATCATGAGCTTGTGCCTCCTC
*lrgB*	F	GCATCGTATCATCGGAGGTA
	R	GGTAACGCAATCGCTGTAGT
*cidA*	F	ATATTGGCACAGAAATTCAAAAGA
	R	TCATAAGCGTCTACACCTTTACGA
*srrB*	F	CGCTTGCCATTGTCCTTGAT
	R	CTTGGTCCATGCGATCCATA
*spa*	F	AAGAAGACGGCAACAAGCCT
	R	AGGCTTGTTGCCGTCTTCTT
*nuc*	F	ATGGACGTGGCTTAGCGTAT
	R	TAGCCAAGCCTTGACGAACT
*pyk* ^a^	F	AATGGTTGCACGTGGTGACA
	R	TAGATTGCGTTGGCAACGTC

*: pyk was used as the internal reference gene.

**Table 2:** Expression fold changes of *S. aureus* ST1792 biofilm related genes in berberine

Category	Gene	Annotation	Log_2_ (Fold-change)
Biofilm			
PIA	*icaA*	poly-beta-1,6-N-acetyl-D-glucosamine synthase	2.22
	*icaB*	biofilm PGA synthesis lipoprotein PgaB	1.85
	*icaR*	TetR/AcrR family transcriptional regulator	−2.32
	*spxA*	regulatory protein spx	−1.42
	*srrB*	sensor histidine kinase ResE	1.08
Protein			
	*fnbA*	fibronectin-binding protein A	1.53
	*fnbB*	fibronectin-binding protein B	1.51
	*spa*	immunoglobulin G-binding protein A	2.60
	*sasG*	surface protein G	2.22
eDNA			
	*nuc*	thermonuclease	2.02
	cidA	holin-like protein	2.06
	*lrgA*	murein hydrolase regulator LrgA	−2.59
	*lrgB*	hydrolase activity	−2.71
Biofilm regulators			
	*agrABCD*	accessory gene regulators	–
	*sigB*	RNA polymerase sigma-B factor	–

### Statistical analysis

At least three independent replicates of each 96-well plate experiment were performed. The statistical analyses were conducted using Student’s t-test with SPSS 24.0(SPSS, IBM, USA). The data were expressed as the means ± standard deviation. Values of *p* < 0.05 were considered statistically significant.

## Results

### The MIC values to berberine of 18 *S. aureus* isolates varied among different MLST types

The MLST test showed that **18** PJI-related *S. aureus* isolates fell into eleven sub-types, with **1** each into ST15, ST17, ST188, ST39, ST1792, ST88, ST8, **2** into ST1281 and **3** into ST630, ST7, ST239 (Table 3), respectively. Among the **11** *S. aureus* sequence types, MIC values of berberine varied from 64 to 512 μg/ml. The highest MIC value was 512 μg/ml for ST 39 and the lowest was 64 μg/ml for ST239. The other **9** MLST types of *S. aureus* displayed the same berberine MIC value of 12 μg/ml (Table 3).

**Table 3:** MLST and Berberine MIC values of 18 clinical S.aureus isolates

MLST Type	Series number	MIC (μg/ml)
ST 15	A	128
ST 17	B	128
ST 188	C	128
ST 1281	D, H	128
ST 39	E	512
ST 630	F, P, Q	128
ST 1792	G	128
ST 7	I, J, K	128
ST 239	L, M, N	64
ST 88	O	128
ST 8	R	128

### Berberine exerted excellent inhibiting effect on PJI-related *S. aureus* strains in planktonic form

**Fig. 1:**
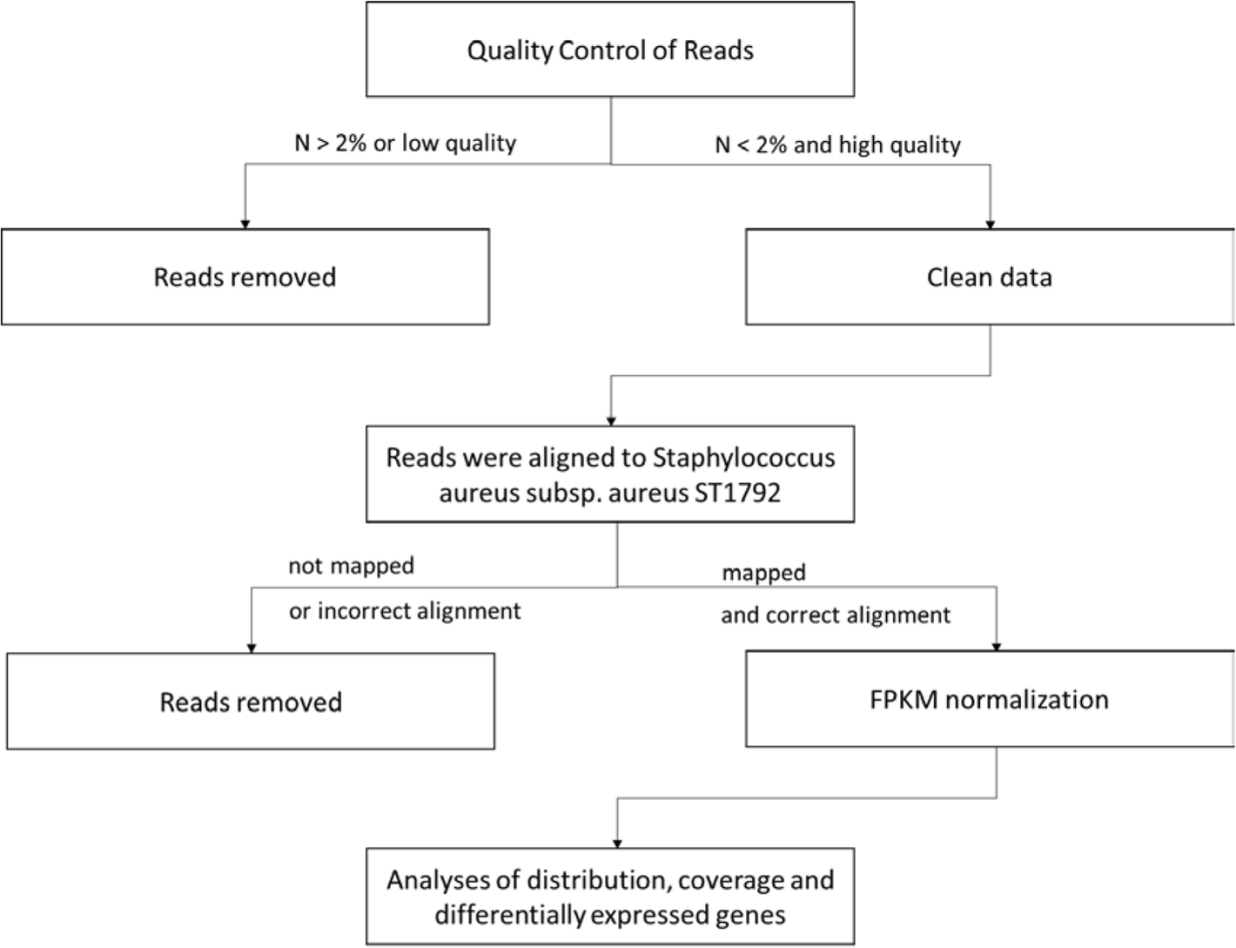
Workflow of transcriptome assembly and annotation for Staphyloccocus aureus

Within 2 h of incubation, no significant growth of all tested *S. aureus* was detected (Fig. 1a and b). After 6 h of incubation, all tested strains showed an essential decrease in the number of bacteria (evidenced by OD value changes) when compared to control group (1% DMSO, Fig. 1c). After 12 h of incubation, within the range of berberine concentration from 32 to 512 μg/mL, a dramatic reduction in the number of bacteria was detected and a total growth inhibition was observed in some strains (Fig. 1d). After 24 h of the study, though some growth could be seen, substantial decreases were noticed in the number of bacteria within the range of berberine concentration from 32 to 512 μg/mL. At the concentrations of 256 and 512 μg/mL, growth of some strains was completely inhibited as the OD (Optical Density) value showed no change (Fig. 1e). The data from *S. aureus* ST39 showed that for all tested berberine concentrations lower than 512 μg/mL, this strain displayed substantial growth which indicated a relatively resistance to berberine compared to the other strains.

### Sub-inhibitory concentrations of berberine promoted biofilm formation in some PJI-related *S. aureus* strains

**Fig. 2:**
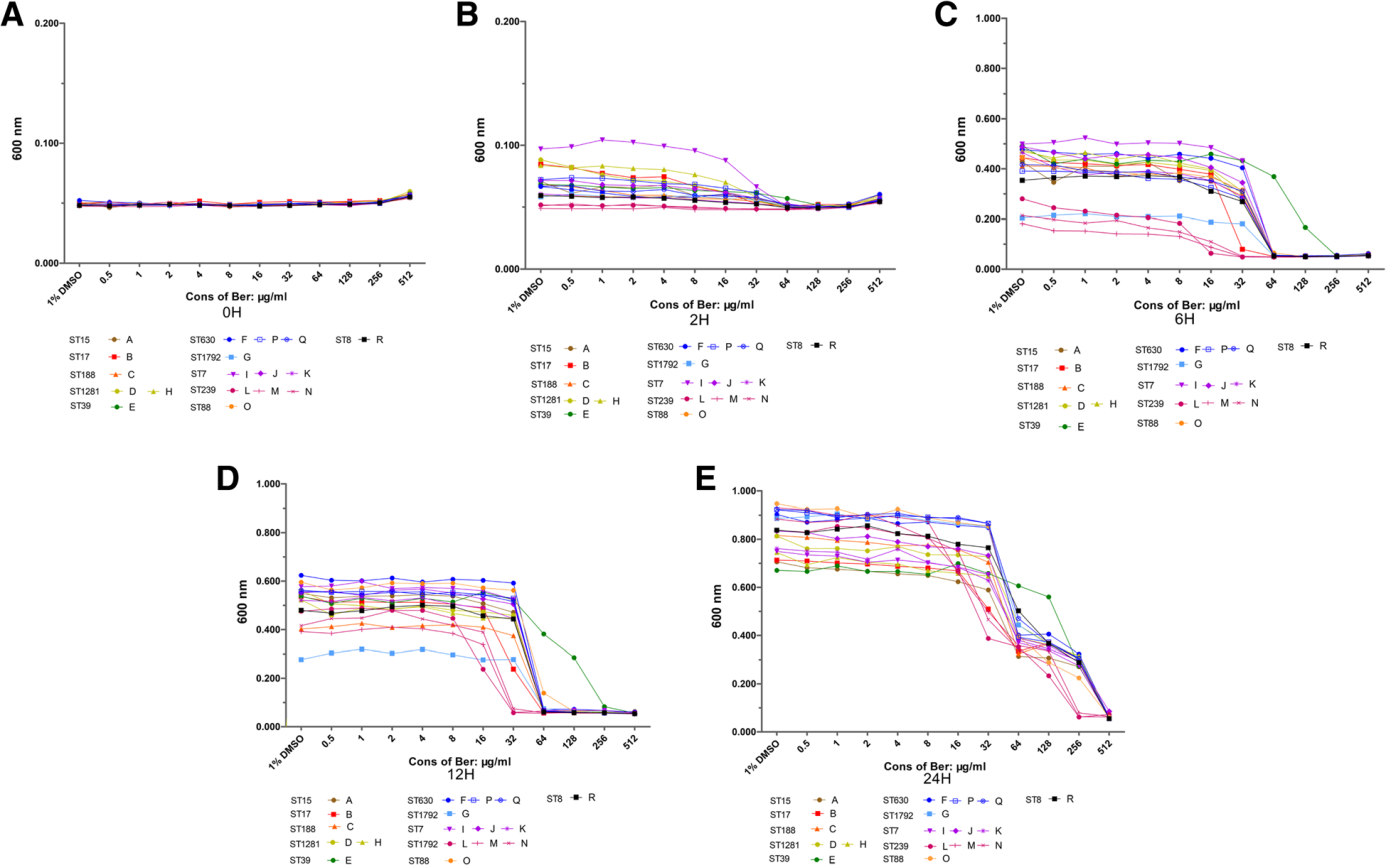
Growth kinetics of clinical S.aureus in the presence of different berberine concentrations after incubation of 0 h (**a**); 2 h (**b**); 6 h (**c**); 12 h (**d**) and 24 h (**e**)

Biofilm assay showed that for all 18 PJI related *S. aureus* strains, berberine yielded excellent anti-biofilm effect at concentrations of MIC or higher as the biofilm biomass was significantly lower than the control group (Fig. 2). However, at low concentrations, the anti-biofilm effect of berberine worked in two distinct modes. For the strains in group 2 (Fig. 2), the biofilm formation reduced as the concentration of berberine increased, which was in accordance with the findings of previous study [30]. However, for the strains in group 1 (Fig. 2), the biofilm formation surprisingly increased along with the concentration of berberine until meeting a sharp drop at 64 μg/ml (ST15, ST17, ST188 and ST630) or 32 μg/ml (ST1281 and ST39) indicating that low concentration of berberine enhanced biofilm formation of these *S. aureus* strains.

### Berberine greatly impacted on global gene transcription of *S. aureus* ST1792

**Fig. 3:**
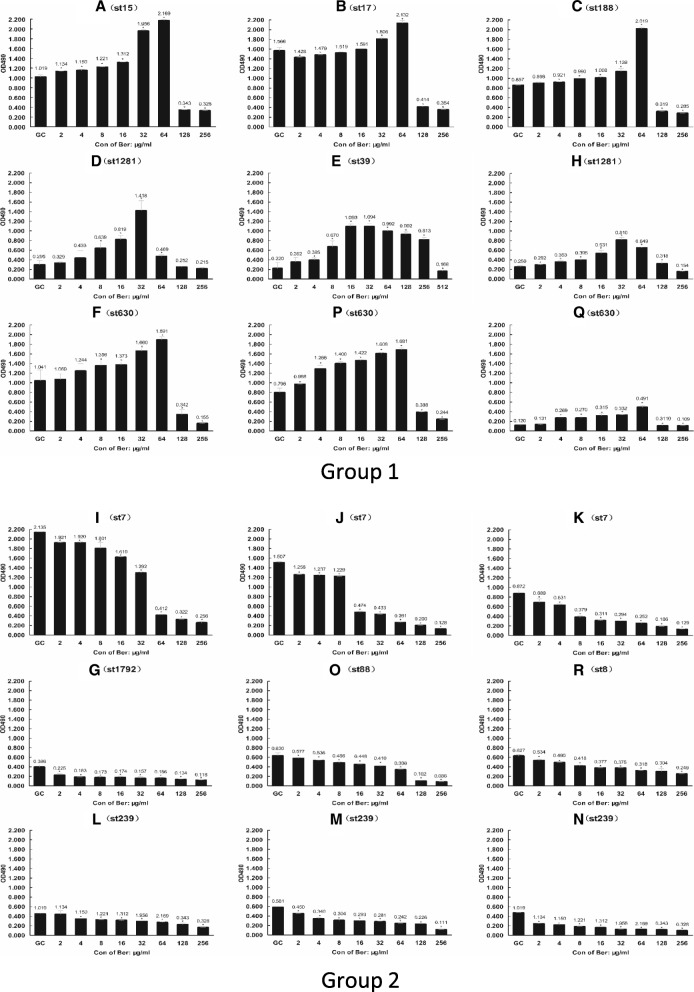
Inhibitory effects of berberine on clinical S.aureus biofilm formation. *S. aureus* strains were inoculated into TSB and cultured with different concentrations of berberine. Wells containing 1% DMSO and bacteria was the bacterial growth control (GC). Data are presented as mean ± standard deviation. *Significance was determined at *P* < 0.05 with comparison to the control group

Transcriptomics analysis reveal the 795 significantly differentially expressed genes in *S. aureus* ST1792 treated with berberine, with 336 genes being up-regulated and 459 down-regulated (Fig. 3a). Volcano-plot map visualized the distribution of 2596 detectable differentially expressed genes, with the red plots representing 336 significantly up-regulated *(log*_*2*_ *Fold-change ≥ 1 or P-value ≤ 0.05)* genes, blue plots representing 459 significantly down-regulated *(log*_*2*_ *Fold-change ≥ 1 or P-value ≤ 0.05)* genes and grey representing 1801 non-significantly regulated *(log*_*2*_ *Fold-change < 1 or P-value > 0.05)* genes (Fig. 3b).

### Berberine induced great differentially expressions of major pathogenic genes in *S. aureus* ST1792

**Fig. 4:**
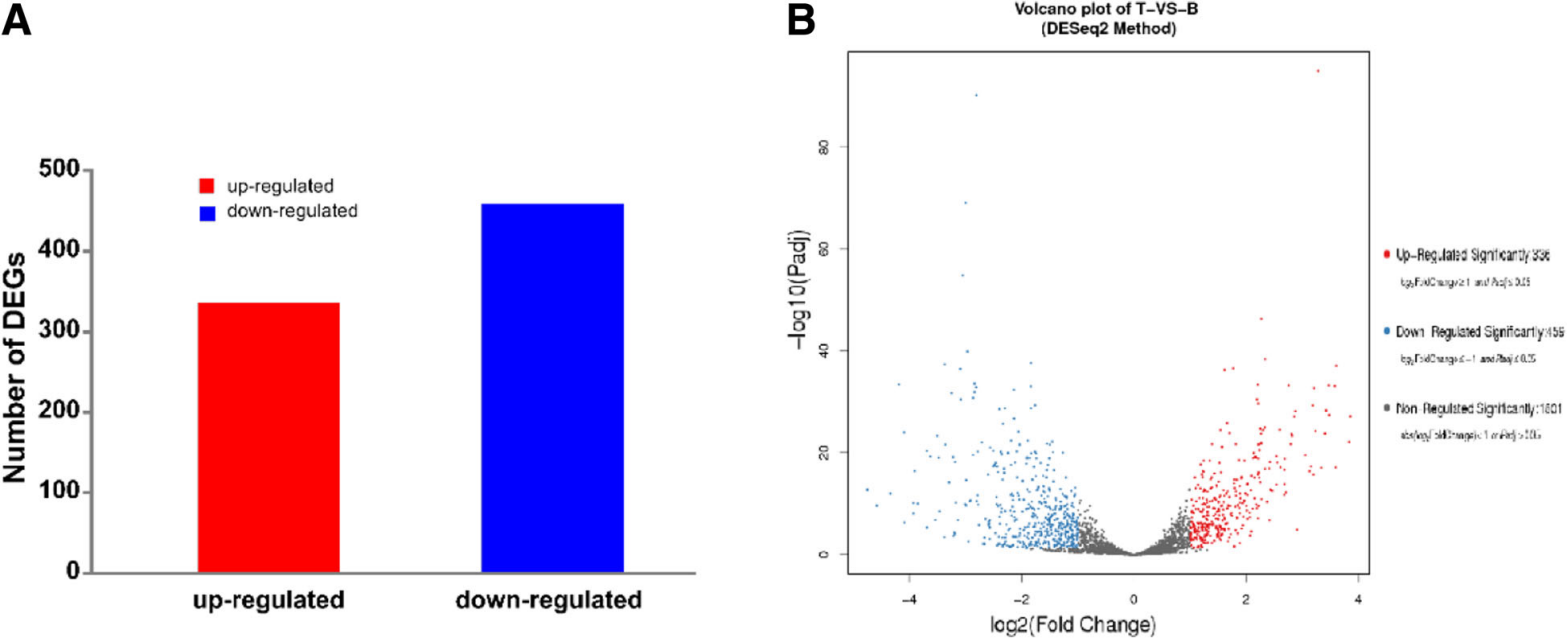
Differentially expressed genes in berberine group compared to TSB control group. **a** Totally 795 significantly differentially expressed genes were detected, of which 336 were up-regulated and 459 were down-regulated (Significance: log2 Fold-change≥1 and *P*-value < 0.05). **b** Volcano-plot map of 2596 detectable differentially expressed genes Red: significantly up-regulated genes; blue: significantly down-regulated genes, grey: non- significantly regulated genes (log2 Fold-change < 1 or *P*-value> 0.05). Wells containing 1% DMSO and bacteria was the bacterial growth control (GC)

The pathogenesis of *S. aureus* in PJI is modulated by the expressions of its abundant pathogenic genes. Transcriptome sequencing revealed the changes in the expressions of major pathogenic genes in *S. aureus* ST1792 caused by berberine, as listed in Table 2 and Fig. 4. The biofilm of *S. aureus* is composed of different matrix including PIA (Polysaccharide Intercellular Adhesin), proteins and eDNA (Extracellular DNA) [37–40] involving different genes, respectively. Unexceptionally, the expressions pf positive regulators (*icaA/B* [41, 42]*, srrB* [43]*, fnbA/B, spa* [44]*, sasG* [45]*, nuc* [46]*, cidA* [47]) of Staphylococcal biofilm were all up-regulated while negative regulators (*icaR* [48]*, spxA* [49]*, lrgA/B* [50]) down-regulated. Notably, two important biofilm regulator of *S. aureus*, the *agr* operon [51, 52] and *sigB* [53–55], were not differentially expressed in berberine.

### RNA-seq results were verified by qRT-PCR

The Log2 (Fold-change) of gene expression in berberine treatment group compared to control group revealed by RNA-seq and qRT-PCR are shown in Table 4. Although there is a slight difference in the exact fold change for each gene between qRT-PCR and RNA-seq, the differences were minor and the gene expression shares a similar trend in qRT-PCR with RNA-seq, which suggested the relatively high consistency between RNA-seq and qRT-PCR.

**Table 4:** The fold-change determined by RNA-seq and qRT-PCR of selected genes

Gene Name	Gene Annotation	Log2 Fold-change	
		RNA-seq	qRT-PCR
*icaA*	poly-beta-1,6-N-acetyl-D-glucosamine synthase	2.22	2.12
*icaR*	Biofilm operon icaADBC HTH-type negative transcriptional regulator IcaR	−2.31	−2.45
*fnbA*	Fibronectin-binding protein A	1.53	1.66
*lrgA*	murein hydrolase regulator LrgA	−2.59	− 2.42
*lrgB*	hydrolase activity	−2.71	−2.51
*cidA*	Holin-like protein CidA	2.06	2.15
*srrB*	sensor histidine kinase	1.08	1.17
*spa*	immunoglobulin G-binding protein A	2.60	2.72
*nuc*	thermonuclease	2.02	2.10

## Discussion

The *S. aureus* is leading pathogen of prosthetic joint infection, imposing great challenges for PJI control, especially with the increasing occurrence of multi-drug resistant *S. aureus* recently due to inappropriate use of antibiotics [56]. Thus, species identification of the pathogen is widely acknowledged the key to right use of antibiotics and avoidance of drug resistance and have been strongly addressed in the clinical practice of orthopedic surgeons. However, it has been found that within the same species of bacteria, subtype strains could present distinct sensitivities to antimicrobials which hasn’t drawn enough attention in clinical practice. As berberine has been proved affective inhibiting wide range of microbes including *S. aureus* [30, 57], it holds a great potential to be a complementary antimicrobial agent for *S. aureus* induced PJI control. Our study first tested the sensitivity of eighteen PJI-related *S. aureus* to berberine and particularly investigated the differences among the MLST subtypes of *S. aureus* and. It was found that eighteen PJI relevant *S. aureus* showed eleven MLST subtypes and berberine presented significant antibacterial activity against all eleven PJI-associated *S. aureus* MLST subtypes, surprisingly however, the MIC values of berberine were quite diverse among different MLST types, as the highest MIC value was 512 μg/ml in ST 39 and the lowest was 64 μg/ml in ST239. The great diversity of MIC values among different MLST subtypes of *S. aureus* reveals the importance of determining the MLST subtypes and accordingly choose the appropriate dosage when applying berberine into clinical *S. aureus* induced PJI control.

Growth kinetics test of eighteen PJI-related *S. aureus* in berberine was conducted and significant planktonic growth inhibition was observed. Consistent with MIC test results, the growth kinetics test also revealed a MLST subtype-depend sensitivities to berberine of *S. aureus* relevant to PJI. Notably, after 24 h, all tested *S. aureus* strains except for ST39 showed remarkable growth in berberine concentrations over their MIC values. Similar results were observed when Robert et al. tested the antimicrobial effect of berberine against coagulase-negative staphylococcus strains in 2014 [29]. Giving these, we propose the reason could be that berberine does not exert antimicrobial efficacy against staphylococci by direct killing, but rather, by inhibiting the bacteria. Thus, certain amount of bacteria is able to survive and persist in high berberine concentration environment, and as berberine gradually depletes the survivors regain a favorable environment. This infers that although berberine displays excellent inhibiting effects against *S. aureus* while used alone berberine is not sufficient to control *S. aureus* infections. Therefore, we suggest that berberine be used as an ancillary drug in combination with other antibiotics in *S. aureus* related PJI control, since berberine shows remarkable synergy effects with a wide range of antibiotics [27, 29, 58, 59]. For example, a study by Zuo et al. revealed that berberine significantly lowered the MIC values of a series of antibiotics against *S. aureus* including MRSA [59]. Furthermore, since local host immunity is often impaired in PJI patients [60] and berberine has been proved able to improve host immunity [61–63], it is a promising candidate for clinical PJI control.

Biofilm plays an essential role in the pathogenesis of *S. aureus*-induced PJI [12, 13]. Berberine showed excellent anti-biofilm effect at concentrations of MIC or higher for all tested PJI related *S. aureus* strains, while unexpectedly, at sub-inhibiting concentrations berberine enhanced biofilm formation in a concentration-depend manner for nine strains (ST15, ST17, ST188, ST630, ST1281 and ST39). This was further confirmed by the transcriptome sequencing that at half MIC berberine concentration, the expression of genes responsible for producing three types of biofilm matrix in *S. aureus* were all up-regulated. This is likely to be due to the bacterial adaptation to the highly-stressed environment produced by berberine since under environmental stress, *S. aureus* tend to live in a biofilm-form instead of planktonic-form [64, 65]. This of acting mode of berberine on PJI-related *S. aureus* biofilm formation infers that the concentration of berberine must reach an ‘instant peak’ like an elevator when used in clinic for PJI control since slow concentration growth like an escalator may improve bacterial biofilm formation. However, oral administration of berberine shows poor absorption [66, 67] and berberine is toxic [68] when given through venous injection. Therefore, we recommend berberine be locally administrated when used in PJI control, for instance, contained in bone cement [69] in combination with other antibiotics. As a result, an ‘instant peak’ of berberine concentration is achieved and co-administration of berberine and antibiotics produces synergic antimicrobial effects and immune enhancement [27, 29, 58, 59, 70].

## Conclusion

Our work showed the antimicrobial ability and the MLST type-dependent action mode of berberine against *S. aureus* related to PJI, which might provide reference for future application of berberine for controlling *S. aureus* induced PJI.

## Data Availability

The datasets generated and analysed during the current study are not publicly available due to its future use in our further studies including investigating the mechanisms of inconsistent antimicrobial effects of berberine against different *S. aureus* subtypes based on the RNA-seq profiles but are available from the corresponding author on reasonable request.

## Abbreviations

DEGs: Differentially Expressed Genes
DMSO: Dimethyl sulfoxide
eDNA: Extracellular DNA
GO: Gene Ontology Consortium
MHB II: Mueller–Hinton Broth II
MIC: Minimum Inhibitory Concentration
MLST: Multi-locus Sequence Types
MRSA: Methicillin-Resistant *S. aureus*
OD: Optic Degree
OTC: Over the Counter
PBS: Phosphate Buffered Saline
PIA: Polysaccharide Intercellular Adhesin
PJI: Prosthetic Joint Infection
RNA-seq: RNA sequencing
*S. aureus*: Staphylococcal aureus
TSB: Tryptic Soy Broth
